# Undetected Intraoperative Periprosthetic Femoral Fractures in Patients Undergoing Primary Total Hip Arthroplasty: A Retrospective Case Series and Literature Review

**DOI:** 10.1111/os.13646

**Published:** 2023-01-17

**Authors:** Yubo Liu, Chao Li, Zheng Cao, Xin Wang, Jiaxin Wen, Hangyu Ping, Xiangpeng Kong, Wei Chai

**Affiliations:** ^1^ School of Medicine Nankai University Tianjin China; ^2^ Senior Department of Orthopaedics The Fourth Medical Center of PLA General Hospital Beijing China; ^3^ National Clinical Research Center for Orthopaedics Sports Medicine & Rehabilitation Beijing China

**Keywords:** Case Series, Femoral Stems, Fracture Lines, Intraoperative Periprosthetic Femoral Fracture, Risk Factors

## Abstract

**Objective:**

Periprosthetic fracture in total hip arthroplasty (THA) can be catastrophic, and early detection and appropriate management are vital to the overall prognosis. This study aimed to describe and summarize the features of undetected intraoperative periprosthetic femoral fractures (IPFFs) in primary THA patients and treatment measures and to review the relevant literature.

**Methods:**

We reviewed a total of 6350 primary THAs performed at our institution between January 2013 and December 2020 and screened all IPFFs. Of 138 IPFFs, 24 were undetected and met the inclusion criteria. We recorded and compared basic patient and operative information and measured some parameters to evaluate canal morphologies based on preoperative radiographs. We also compared fracture line characteristics using postoperative radiographs to summarize the features of intraoperative fractures and propose treatment strategies. The Kolmogorov–Smirnov test was used to test the normality of the variable distributions. Measured parameters in all groups were analyzed using one‐way analysis of variance and compared using Dunnett's test. The χ^2^ and Fisher exact tests were used to compare reoperation rates across the groups. Interrater and intrarater reliability were evaluated by intraclass correlation coefficients.

**Results:**

Among the 24 hips, there was no significant difference in patient demographics, basic operative information or morphology. The incidence of IPFFs in primary THA patients was 2.17%, and up to 17.4% of IPFFs were undetected until postoperative fluoroscopy. The incidence of undetected IPFFs among all primary THA patients was 0.38% and varied by stem type, with the highest incidence in femurs with either anatomical (1.04%, 4/385) or modular stems (0.90%, 9/1003). Femurs with anatomical stems had a higher reoperation rate. The distal periprosthetic (Gruen zone 4) fracture line of femurs with tapered stems was more prone to involve the medial or lateral bone cortex, which could cause instability.

**Conclusion:**

An undetected IPFF is most likely in femurs fitted with a prosthesis of an inappropriate size or type. Anatomical stems will most likely cause unstable fractures; thus, it is recommended to use them with caution and note the possibility of medial distal femoral fracture. Improper modular stem type or size selection results in longitudinal fractures of the distal femur, and prophylactic cerclage wire binding is recommended in dysplastic hips. Incorrect use of tapered stems in well‐ossified femurs may cause distal femoral fractures involving the medial or lateral bone cortex. Intraoperative fluoroscopy after implantation may help detect hidden fractures.

## Graphical abstract

IPFF is a severe complication of THA and sometimes catastrophic. To provide reference and guidance for the clinical problems, we reviewed all the cases of undetected IPFFs in patients undergoing primary THA at our institution and the related literature regarding this issue, aiming to (1) describe and summarize the features of IPFFs, (2) identify potential risk factors and (3) propose possible early detection measures and strategies to avoid more serious outcomes.

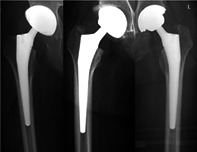

## Introduction

Periprosthetic fracture is a severe complication of total hip arthroplasty (THA) and is sometimes catastrophic. It has become a major reason for revision surgery, accounting for 6% of revisions recorded in registries in the United States and 20% in Australia^1^; in particular, periprosthetic femoral fracture (PFF) is an increasingly prominent cause, resulting in poor clinical outcomes and high mortality rates.^2^, ^3^, ^4^, ^5^

Studies have shown that the incidence of intraoperative periprosthetic femoral fractures (IPFFs) is not less than that of postoperative periprosthetic femoral fractures.^6^, ^7^ Fractures identified intraoperatively can be treated during primary surgery and have statistically significantly better treatment outcomes and a lower rate of catastrophic consequences than fractures identified postoperatively.^2^, ^7^ Those fractures that are undetected and untreated intraoperatively may result in delayed weight bearing and returning to normal activities, which sometimes need difficult and controversial management strategies and necessitate complicated and serious reoperations.^8^ Implant survival is dependent on a stable bone‐prosthesis interface, so it is crucial to identify fractures that disrupt the femoral‐implant interface because failure in recognition could result in catastrophic consequences for prosthesis performance and survival.^9^

IPFFs have become more common in recent years due to the growing use of cementless press‐fit implants, whereas postoperative fractures appear to be linked to an overall increase in the at‐risk population undergoing arthroplasty.^10^, ^11^ However, it has been reported that several characteristics of patients are also linked to an increased risk of intraoperative periprosthetic fractures, such as female sex, advanced age, osteoporosis, and poor bone quality.^12^, ^13^ Other risk factors include use of the direct anterior approach (DAA) in surgery, stem broaching with a multiple‐toothed stem broach,^14^ and implantation of double wedge metaphyseal filling (“fit‐and‐fill”) and anatomical femoral stems.^15^ However, there have been no relevant large cohort studies in which researchers compared the clinical characteristics of or risk factors or countermeasures for IPFFs in patients undergoing primary THA between different demographic groups or different prosthesis types. Therefore, it is necessary to summarize the characteristics of undetected IPFFs and put forward preventive measures.

The purpose of the study was to provide a reference and guidance for the above clinical problems. We reviewed all the cases of undetected IPFFs in patients undergoing primary THA at our institution and the related literature regarding this issue, aiming to (1) describe and summarize the features of IPFFs, (2) propose possible early detection measures and strategies to avoid more serious outcomes.

## Methods

### Patients

**FIGURE 1:**
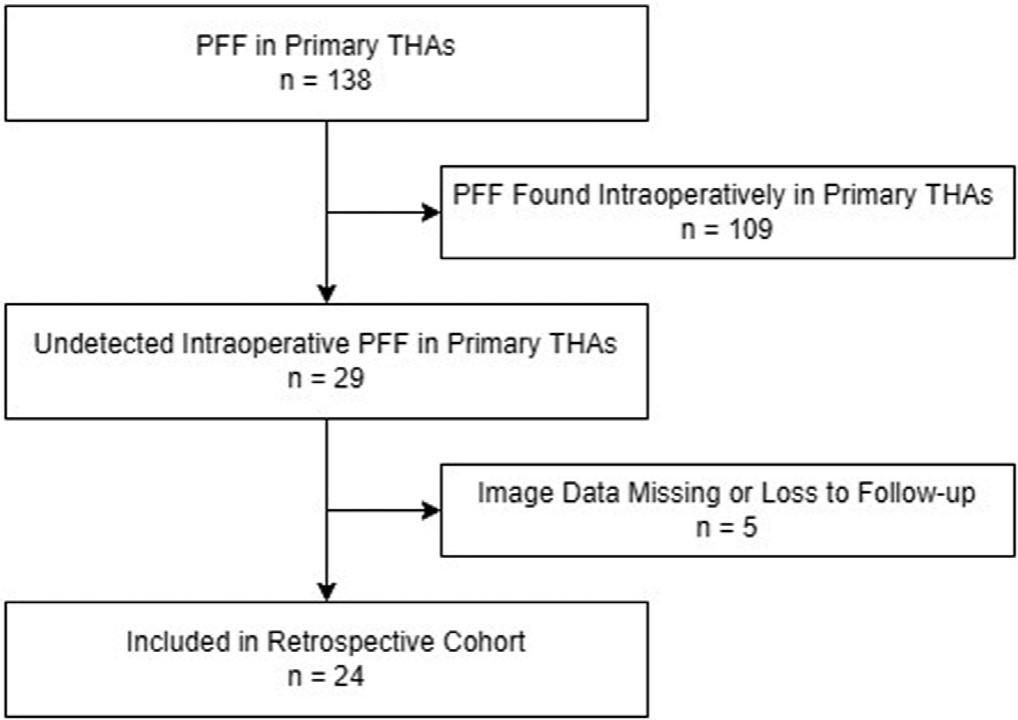
Flow chart of the case selection

We reviewed all PFFs that occurred in a total of 6350 primary THAs performed at our institution from January 2013 to December 2020 and retrospectively studied the PFFs that were sustained during the operation but were not detected intraoperatively in patients of any age or either sex (Figure 1).The study was approved by the ethics committee of Chinese PLA General Hospital (S2020‐005‐01). Patients (1) with preoperative and postoperative anteroposterior (AP) and lateral pelvic radiographs; (2) with an IPFF sustained during primary THA with a clear diagnosis confirmed *via* X‐ray or CT; (3) with no intraoperative detection or treatment; and (4) who were followed up until the fracture was completely healed were included. Patients (1) with no complete clinical data; (2) undergoing revision THA; (3) with fractures found intraoperatively, with or without corresponding treatment; or (4) lost to follow‐up before fractures were healed were excluded.

All operations were performed at our institution, and the main types of prostheses and the corresponding indications were as follows. Modular stems (S‐ROM, DePuy, USA) were implanted for patients with Crowe III or IV developmental dysplasia of the hip (DDH), Crowe I and II DDH with excessive abnormal femoral anteversion and those with deformities of the proximal femur. Tapered stems (Corail, DePuy, USA and LCU, Link, Germany) were used for Dorr B and C non‐DDH patients. Anatomical stems (Ribbed, Link, Germany) were the preferred choice in early years, and bone preservation stems (Tri‐lock, DePuy, USA) were used in femurs with relatively abundant cortical bone (non‐Dorr C femurs). In the 6350 hips, there were 1003 modular stems, 2544 tapered stems, 385 anatomical stems, and 225 bone preservation stems. The corresponding IPFF incidence was calculated according to the total number of fractures associated with each stem type.

The preoperative two‐dimensional surgical plan was made using traditional film and manual measurement (early years) or OrthoView software (version 6.6.1, Materialize, Leuven, Belgium) (recent years). All operations were performed under single general anesthesia with the patient in the lateral decubitus position. All procedures were routinely performed *via* a posterolateral approach. The follow‐up time at which fracture union was completed was considered the healing time.

### Clinical Assessment and Radiographic Measurement

**FIGURE 2:**
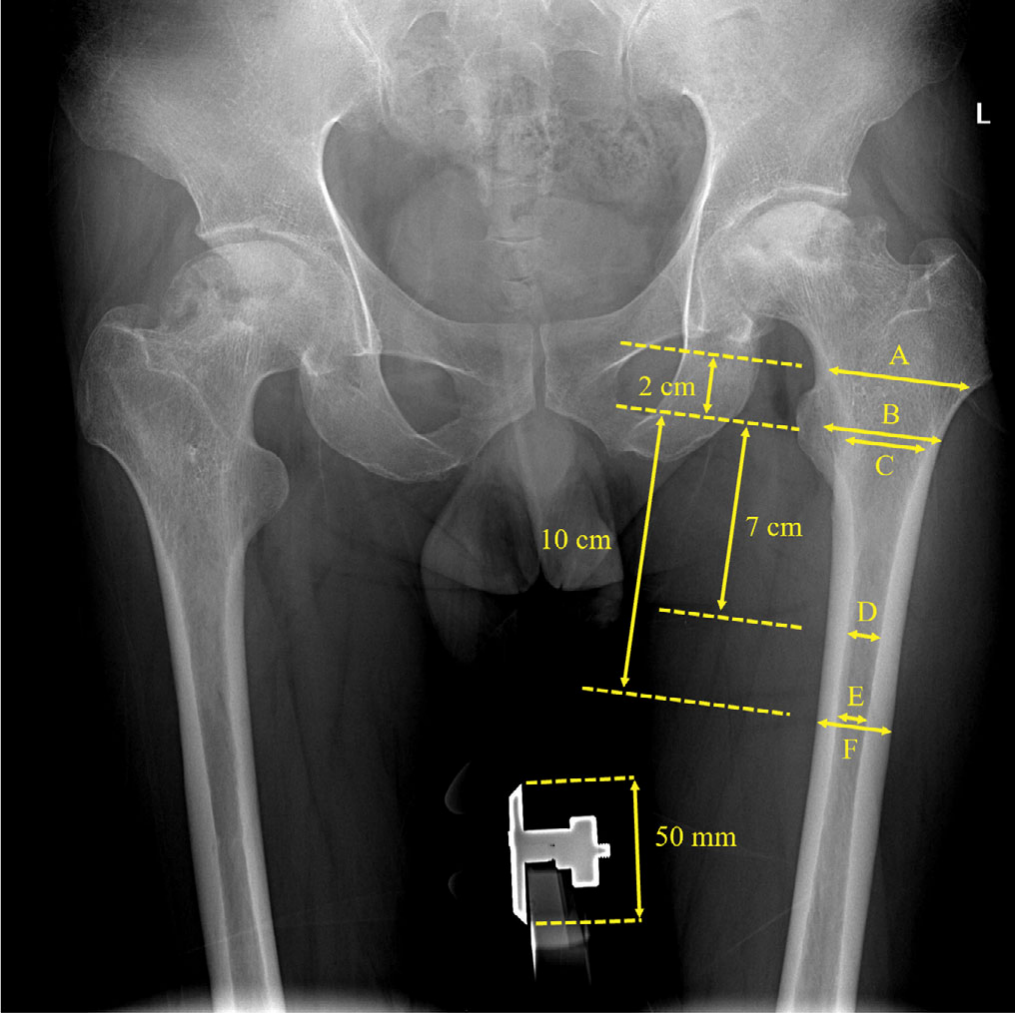
CCR, canal‐calcar ratio = E/C; CFI, canal flare index = A/E; MCI, morphological cortical index = B/D; CBR, canal bone ratio = E/F

The reason for surgery, number of previous operations at the affected site, intraoperative condition, and type of femoral prothesis were obtained from the patients' medical records. The morphology of the proximal and middle femoral medullary cavities was evaluated by radiological indices that are commonly used to describe the degree of osteoporosis. According to the model proposed by Yeung et al.,^16^ the canal width (lesser trochanter) (line B), calcar isthmus width (lesser trochanter) (line C), canal width (lesser trochanter +20 mm) (line A), isthmus width (mediolateral, lesser trochanter +70 mm) (line D), isthmus width (mediolateral, lesser trochanter +100 mm) (line E), and extracortical width (mediolateral, lesser trochanter +100 mm) (line F) were marked and measured by referring to a standardized marker routinely used at our institution (Figure 2). We evaluated the canal‐calcar ratio (CCR), canal flare index (CFI), morphological cortical index (MCI), and canal bone ratio (CBR) to assess bone quality and divided the canals into groups according to the Dorr classification^16^, ^17^, ^18^, ^19^, ^20^ by integrating the above measurements.

**FIGURE 3:**
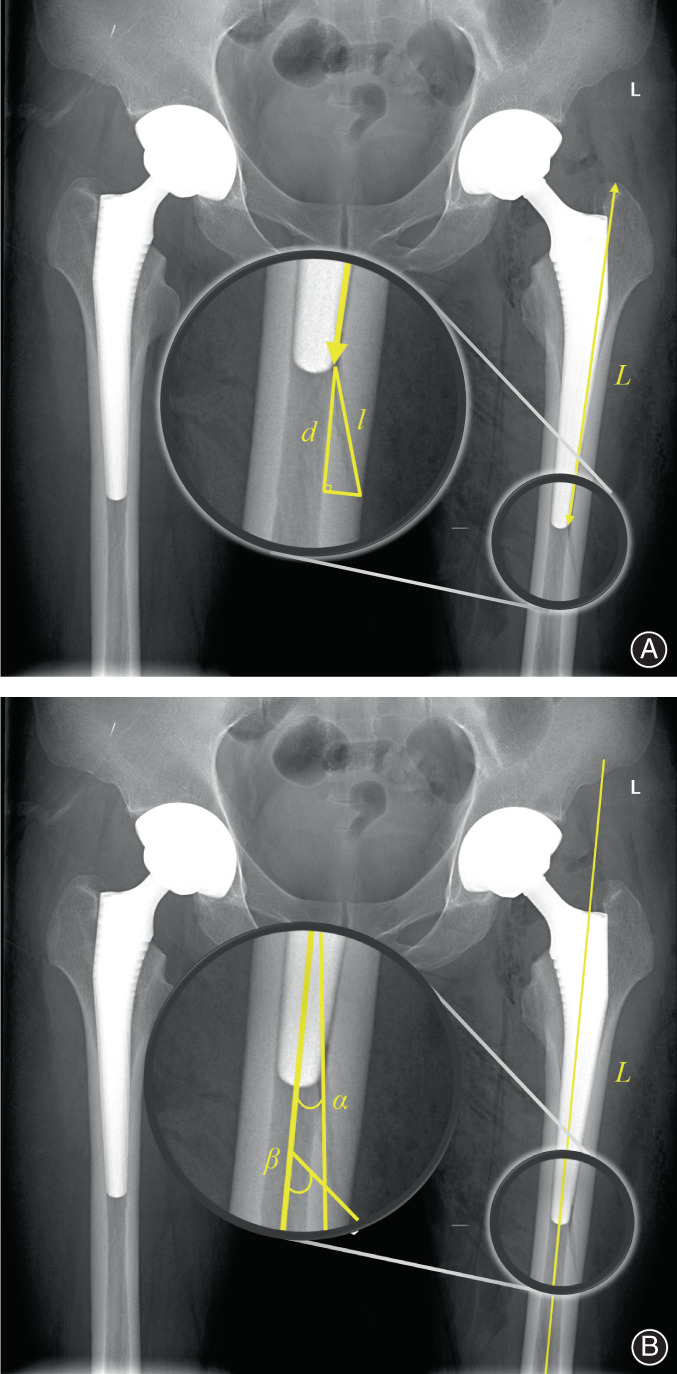
(A) Measurement of the distance from the origin of the fracture line appearance to the tip of the greater trochanter (*L*), length of the fracture line appearance (*l*), and vertical distance of the fracture line appearance (*d*). (B) Measurement of angle formed by the tangent of the origin of the fracture line appearance to the long axis of the femur (*α*) and angle formed by the tangent of the distal endpoint of the fracture line appearance to the long axis of the femur (*β*)

On the basis of postoperative AP radiographs, we divided the fracture lines into seven regions according to Gruen's femoral zones classification.^21^ In addition, for fractures in zone 4 that reached the medial or lateral cortical bone on AP radiographs, access to the medial cortex was defined as 4(a), and lateral access was defined as 4(b). We also measured some important parameters to describe the fracture lines, as follows: distance from the origin of the fracture line appearance to the tip of the greater trochanter (*L*); length of the fracture line appearance (*l*); vertical distance of the fracture line appearance (*d*); angle formed by the tangent of the origin of the fracture line appearance to the long axis of the femur (*α*); and angle formed by the tangent of the distal endpoint of the fracture line appearance to the long axis of the femur (*β*) (Figure 3).

All radiographic parameters were measured twice over a 2‐week period by two independent investigators using the Medcare Imaging System (Medcare Co., Ltd., Qingdao, Shandong, China). The arithmetic mean values of the four measurements were used for statistical analysis.

### Statistical Analysis

Descriptive data are expressed as the mean with SD or frequency and percentage. The Kolmogorov–Smirnov test was used to test the normality of the variable distributions. The measured parameters in all groups were analyzed using one‐way analysis of variance and compared using Dunnett's test. The χ^2^ test and Fisher exact test were used to compare reoperation rates across the groups. Interrater and intrarater reliability were evaluated by intraclass correlation coefficients (ICCs). A *p* value <0.05 was considered statistically significant. Statistical analyses were performed using SPSS 25.0 (IBM Corp, Armonk, NY, USA).

## Results

### Demographics and Incidence of Undetected IPFFs

A total of 138 PFFs were sustained in patients who underwent primary THA between January 2013 and December 2020, and in 23 patients, 24 undetected IPFFs met the inclusion criteria and were retrospectively reviewed. The demographics of the patients are shown in Table 1. Among the patients, eight of 24 (33.3%) had a history of previous surgery on the hip in which the IPFF occurred. The 24 hips were measured and divided into four groups by femoral stem type: anatomical stems (Ribbed, Link, Germany), n = 4; modular stems (S‐ROM, DePuy, USA), n = 9; tapered stems (Corail, DePuy, USA and LCU, Link, Germany), n = 10; and bone preservation stems (Tri‐lock, DePuy, USA), n = 1.

**TABLE 1:** Patient demographics and basic operative information

No. of patients	23
Mean age (years) (mean ± SD)	46.8 ± 14.6
Sex (male/female)	12/11
BMI (kg/m^2^) (mean ± SD)	23.4 ± 4.4
No. of hips	
Left	11
Right	13
Diseases	
DDH	13
ONFH	8
AS	2
OA	1
No. of previous operations	
0	16
1	6
2	1
4	1
Operating time (min) (mean ± SD)	113.7 ± 34.5

Abbreviations: AS, ankylosing spondylitis; BMI, body mass index; DDH, developmental dysplasia of the hip; OA, osteoarthritis; ONFH, osteonecrosis of the femoral head; SD, standard deviation.

**FIGURE 4:**
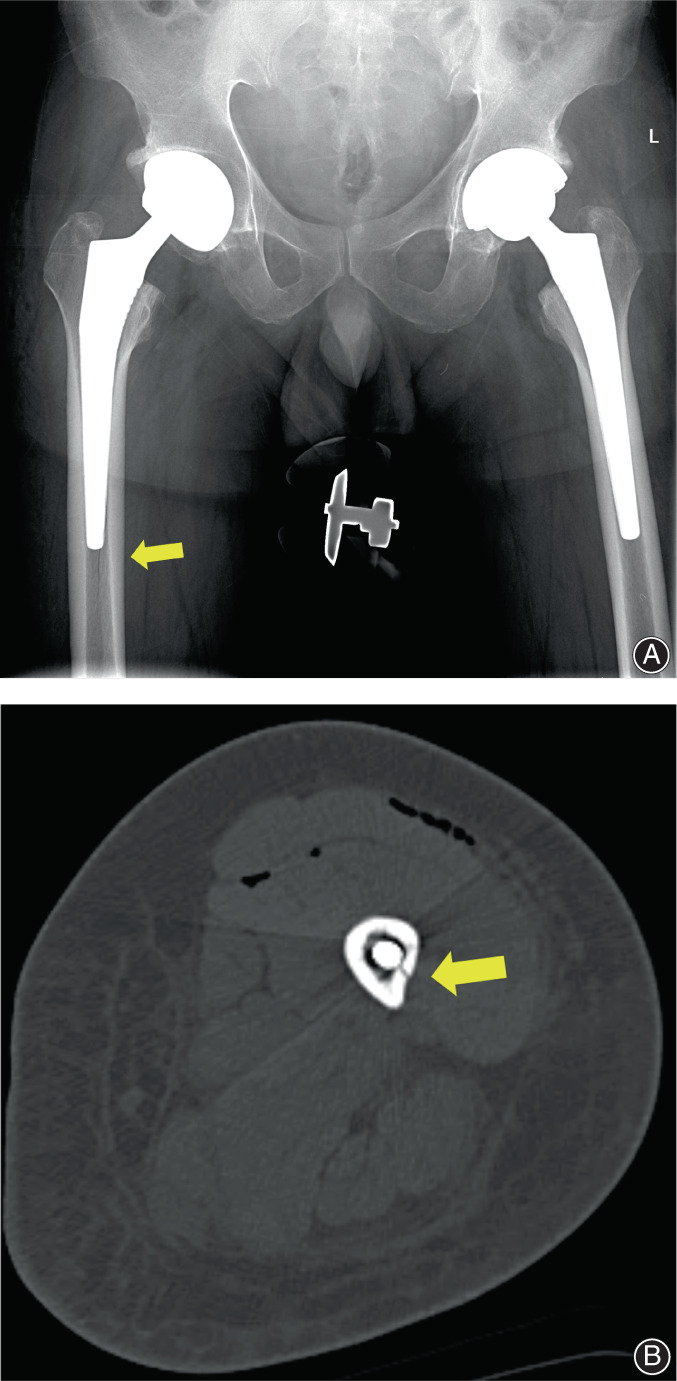
(A) A 52‐year‐old male underwent THA due to ONFH. Postoperative radiograph showed a longitudinal extended fracture at the distal side of the right femur. The fracture healed 3 months postoperatively without reoperation. (B) A 43‐year‐old female underwent THA due to DDH. Postoperative CT showed a fracture of the distal femur around the modular prosthesis. Fracture healing was observed at the 3‐month postoperative follow‐up without reoperation

According to our statistical results, the incidence of IPFFs in all primary THA patients was 2.17% (138/6350), and up to 17.4% (24/138) of IPFFs were undetected until postoperative fluoroscopy or CT (Figure 4). The incidence of IPFFs was 1.04% (4/385) for anatomical stems, 0.90% (9/1003) for modular stems, 0.39% (10/2544) for tapered stems, and 0.44% (1/225) for bone preservation stems.

### Interobserver and Intraobserver Reliabilities

All the ICCs for interobserver and intraobserver reliability of the measurements were greater than 0.75 (*p* < 0.01), respectively. Therefore, the ICCs for interrater and intrarater reliability indicated strong correlations.

### Measurement of the Medullary Cavities

The mean CCR was 0.41 ± 0.07, the mean CFI was 4.11 ± 1.10, the mean MCI was 3.01 ± 0.58, and the mean CBR was 0.46 ± 0.10. Through comprehensive analysis of the results, the morphology of the medullary cavity was classified according to the Dorr classification. The results, prosthesis types, Gruen zone results, and fracture outcomes are shown in Appendix S1 (Supplementary File). Most fracture lines were in Gruen zones 3 to 5 (87.5%), indicating that the fracture lines were mostly located distal to the prosthesis. The mean healing time was 7.13 months.

### Characteristics of Fracture Lines and Rates of Reoperation

For all 24 fractures, the mean values of *L*, *l*, *d*, *α*, and *β* in each subgroup are shown in Table 2 (the one case in which a bone preservation stem was used was excluded because of the small sample size). No significant differences were found. Femurs with anatomical stems had a higher rate of reoperation (75.0%) than femurs with the other two types (10.0% and 11.1%, respectively).

**TABLE 2:** Characteristics of fracture lines and results of reoperation

Parameters	Overall (n = 23)	Anatomical stems (n = 4)	Tapered stems (n = 10)	Modular stems (n = 9)	*F* value	*p* value
*L* (mm)	147.34 ± 30.83	130.44 ± 7.83	154.99 ± 27.12	146.34 ± 12.82	0.905	0.420
*l* (mm)	31.27 ± 18.14	46.98 ± 12.20	27.00 ± 10.71	29.03 ± 6.67	2.015	0.159
*d* (mm)	29.83 ± 18.31	46.22 ± 12.12	25.32 ± 11.01	27.55 ± 6.66	2.190	0.138
*α* (°)	10.95 ± 8.82	10.2 ± 2.3	10.47 ± 4.64	11.8 ± 4.5	0.067	0.935
*β* (°)	26.30 ± 20.44	36.4 ± 12.2	22.68 ± 20.88	25.8 ± 6.4	0.623	0.546
No. of Reoperations	7 (30.4%)	3 (75.0%)	3 (30.0%)	1 (11.1%)	4.756	0.067

## Discussion

At our institution, the incidence of undetected IPFFs in all primary THA patients was 0.38% (24/6350), and the reoperation rate was 30.4% (7/24). The incidence of undetected IPFFs was the highest for anatomical stems (1.04%, 4/385) and modular stems (0.90%, 9/1003), while the incidence of undetected IPFFs for tapered stems (0.39%, 10/2544) and bone preservation stems (0.44%, 1/225) was approximately equal to the overall average (0.38%, 24/6350). Early recognition and proper treatment of such fractures is vitally important. Therefore, we summarize and discuss the characteristics of and possible risk factors for such fractures to reduce their incidence and prevent them from causing more profound damage in the future.

### Patient‐ and Bone Quality‐Related Factors

In some studies, researchers have discussed some factors that increase the incidence of IPFFs (Table 3). In our case series, the mean age was 46.8 ± 14.6 (range: 23 to 70) years, and the female sex accounted for 47.8% of patients. Up to 33.3% of patients had a history of previous surgery on the affected hip, suggesting it as a risk factor due to the effect of postoperative stress changes. Several radiological indices are used to indicate osteoporosis and bone quality. The CCR reported for patients with osteoporosis ranges from 75% to 100%.^18^ When subjective descriptions of canal shape were compared to actual CFI values, it was discovered that stovepipe canals were characterized by CFI values less than 3.0, typical canals were characterized by values ranging from 3.0 to 4.7, and champagne‐fluted canals were characterized by values ranging from 4.7 to 6.5.^19^ The MCI was measured to describe the morphology of the proximal femur and used to select the fixation method but was also associated with the bone quality in the proximal femur.^16^ It has been suggested that a cementless femoral component should be used if the MCI is greater than 3.0, while a cemented femoral component should be considered if the MCI is less than 2.3. Yeung et al. proposed use of the CBR, which is determined by quantifying both thinning of the cortices in the proximal femur and widening of the endosteal diameter; unlike the CCR, CFI, and MCI, the CBR is measured at a single level above the isthmus of the medullary canal to avoid the effect of abnormal morphology near the lesser trochanter.^16^ We integrated the above methods and classified each medullary cavity into Dorr types: nine of 24 (37.5%) were Dorr type A; eight of 24 (33.3%) were Dorr type B; and seven of 24 (29.2%) were Dorr type C. None of the subtypes showed a significant advantage in terms of fracture cases.

**TABLE 3:** Literature review of reported IPFF rates and related risk factors

R	Study	*T*	*n*	IPFF Rate	Risk factor(s)
22	Zhang et al.^22^	CC	3912	0.77%	Female sex; diagnosis of DDH; and CBR ≥0.49.
23	Fleischman et al.^23^	CH	3126	1.79%	Premature engagement of the diaphysis before reaching a metaphyseal press‐fit by first‐generation tapered wedge stem.
24	Sershon et al.^24^	CH	6309	0.85%	Female sex; age > 65 years; and BMI <25.
25	Cohen et al.^25^	CC	487	2.46%	Dorr B bone.
26	Hartford et al.^26^	CS	500	2.60%	DAA in female patients; patients with morbid obesity (BMI > 40); small Dorr ratio; and small implant size.
27	Zhao et al.^27^	CC	904	2.65%	Corail femoral stem; anterolateral surgical approach; advanced age; and a low Metaphyseal‐Diaphyseal Index score.

Abbreviations: CC, case–control study; CH, cohort study; CS, case series; *n*, number of patients; R, reference number; *T*, type of study.

### Prosthesis‐ and Femur Morphology‐Related Factors

**FIGURE 5:**
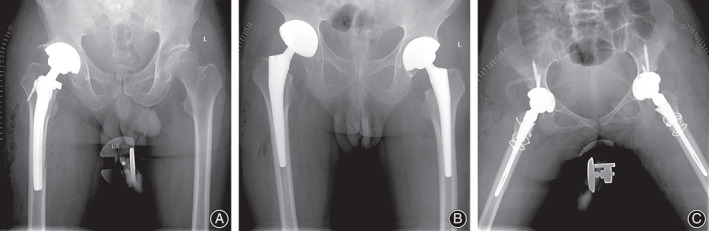
(A) A 65‐year‐old male underwent THA with a ribbed stem because of hip dysplasia and was found to have sustained an IPFF at the distal side of the right femur the day after surgery. No reoperation was performed because the fracture was regarded as stable. The patient was asked to delay weight‐bearing and received intensive care. The fracture was found to be healed at a subsequent follow‐up. (B) A 62‐year‐old male underwent one‐stage bilateral THA with a Corail stem because of hip fusion resulting from ankylosing spondylitis. Postoperative AP radiograph showed that the femoral stems did not match the medullary cavities and that a fracture of the distal femur was present on the right side, which had not been detected intraoperatively. The fracture healed 3 months postoperatively, and the patient had a good prognosis without reoperation. (C) A 25‐year‐old female underwent one‐stage bilateral THA with S‐ROM modular stems. Subtrochanteric shortening osteotomies were performed due to high hip dislocations. Postoperative radiograph showed a fracture line of the distal femur on the right side. The prophylactic cerclage wires were bound, reoperation was avoided, and bone union was observed at the 6‐month follow‐up

We also grouped this case series by the type of femoral prosthesis and compared the characteristics of the fracture lines observed on postoperative radiographs between each group (Appendix S1 and Table 2). Three of four fracture lines in femurs with anatomical stems appeared in Gruen zone 5 (75%), indicating that most fractures occurred at the distal medial site of the stems. Among tapered stems, eight of 10 (80.0%) fracture lines were located in Gruen zone 4, half (n = 4) of which could then be classified into 4(a) and 4(b), suggesting involvement of the medial or lateral cortex (represented by a larger *β* value). Among modular stems, six of nine fracture lines appeared in Gruen zone 4, and none of them belonged to 4(a) or 4(b), which means that all the lines were longitudinally extended without involving the medial or lateral cortex. The PFFs caused by anatomical stems can likely be attributed to the substantial proximal femoral geometry variability that can affect the distribution of mechanical stress.^15^ As a result, the fractures in the anatomical stem group were hardly stable, and three of them (75%) required reoperation to achieve stabilization. The causes of IPFFs in dysplastic hips are aberrant proximal femoral anatomy, abnormal valgus neck geometry, and poor bone quality.^20^, ^22^ Among the cases in which S‐ROM modular stems were used, eight of nine (88.9%) underwent prophylactic wire binding before implantation (0% and 10.0% in the other two groups), and one of nine (11.1%) required reoperation, for a lower reoperation rate than that in the other two groups (75.0% and 30.0%) (Figure 5).

### Strategies

To prevent a possible IPFF and to detect hidden IPFFs as early as possible, we offer the following suggestions: (1) Use anatomical stems with caution and note the possibility of fracture of the medial distal femur after implantation. (2) Avoid over‐indicating the use of tapered stems and choose sizes carefully. (3) Pay attention to aberrations in the proximal femoral anatomy, abnormalities in valgus neck geometry, and poor bone quality in dysplastic hips. Prophylactic cerclage wire binding is helpful in cases of hip dysplasia to reduce the risk of reoperation when IPFFs may occur or have occurred. (4) Intraoperative fluoroscopy after implantation is recommended and may help detect hidden fractures.

### Strengths and Limitations

Strengths of this study include the large sample size, the duration of the cohort study, the strict inclusion of primary THAs with all kinds of prostheses, and the independent analysis between observers, which enhanced the scientific quality.

There are several limitations to this study. First, it was a single‐center retrospective study, with inherent flaws and biases. Second, although the follow‐up was completed when fracture union was observed, the follow‐up period was still short. We have limited information regarding longer‐term follow‐ups and the prognosis of those who underwent reoperation. Third, fractures on the acetabular side or occult peri‐hip fractures that required CT detection were not counted or analyzed. Therefore, these aspects should be considered in future studies to draw more precise and robust conclusions.

### Conclusion

According to the results, we can at least draw the following conclusions: (1) The incidence of IPFFs sustained during primary THAs was 2.17%, up to 17.4% of IPFFs remained undetected until postoperative imaging, and 30.4% of them required reoperation. (2) The femurs that were most prone to have undetected IPFFs were not those with osteoporosis or poor bone quality but those fitted with a femoral prosthesis of an inappropriate type or size. (3) Anatomical stems were most likely to cause unstable fractures, while improper modular stem type or size selection resulted in longitudinal fractures in distal femurs. Incorrect use of tapered stems in well‐ossified femurs may cause a fracture of the distal femur involving the medial or lateral cortical bone.

## Authors' Contributions

All authors have made substantial contributions to the following: (1) the conception and design of the study, acquisition of data, or analysis and interpretation of data; (2) drafting of the article or revising it critically for important intellectual content; and (3) final approval of the version to be submitted. Yubo Liu, Chao Li, and Zheng Cao were primarily responsible for all computational analyses in the article, drafting of the manuscript, and revision of the manuscript. Yubo Liu, Chao Li, and Zheng Cao contributed to this work equally and were co‐first authors. Xin Wang, Jiaxin Wen, and Hangyu Ping screened all cases during the decade, performed preliminary and further measurements and recorded the results. Xiangpeng Kong and Wei Chai were primarily responsible for oversight of the research project, including all data acquisition and analysis and manuscript preparation and approval. Xiangpeng Kong and Wei Chai contributed to this work equally, and both were co‐corresponding authors. All authors have read and approved the final submitted manuscript.

## Disclosures

The authors declare that they have no competing interests. No benefits in any form have been received or will be received from a commercial party related directly or indirectly to the subject of this article.

## Supporting information


**Appendix S1.** Dorr classification, femoral prosthesis types and outcomes of fractures
