# Supplementary material for: Undetected Intraoperative Periprosthetic Femoral Fractures in Patients Undergoing Primary Total Hip Arthroplasty: A Retrospective Case Series and Literature Review
Source: Orthop Surg. 2023 Jan 17;15(3):758–65. doi: 10.1111/os.13646 (PMC9977600; doi:10.1111/os.13646)
Supplement: Supplementary file 1 — Appendix S1. Dorr classification, femoral prosthesis types and outcomes of fractures [file OS-15-758-s001.docx]

Appendix 1. Dorr Classification, Femoral Prosthesis Types and Outcomes of Fractures

| Case | Dorr Classification | Femoral Prosthesis Type | Gruen Zone | Reoperation |
| --- | --- | --- | --- | --- |
| 1 | A | Anatomical | 6 | Yes |
| 2 | A | Modular | 4 | No |
| 3 | C | Tapered | 2 | No |
| 4 | B | Tapered | 4 | No |
| 5 | B | Tapered | 4 | No |
| 6 | B | Modular | 4 | No |
| 7 | C | Modular | 4(a) | Yes |
| 8 | A | Tapered | 4(b) | No |
| 9 | B | Tapered | 4 | Yes |
| 10 | C | Modular | 4 | No |
| 11 | C | Anatomical | 5 | Yes |
| 12 | C | Anatomical | 5 | Yes |
| 13 | B | Modular | 4 | No |
| 14 | A | Tapered | 3 | Yes |
| 15 | B | Tapered | 4(b) | Yes |
| 16 | A | Tapered | 4 | No |
| 17 | B | Anatomical | 5 | No |
| 18 | C | Modular | 2 | No |
| 19 | B | Bone preservation | 5 | No |
| 20 | A | Modular | 5 | No |
| 21 | A | Tapered | 4 | No |
| 22 | A | Modular | 5 | No |
| 23 | A | Tapered | 4(b) | No |
| 24 | C | Modular | 4 | No |
